# 

**Published:** 1870-01

**Authors:** 


### NEW BOOKS.

MARK TWAIN'S BOOK is the most popular one that has been published for years. Mr. Geo. W. Dale, with head quarters at *Gazette* office, is agent for this County. He is selling piles of them. As they can be had *only* of agents, those desiring the book can apply as above. "The American Publishing Co.," 149 Asylum Street, Hartford, Conn., want agents in every county in the Union.

"HITCHCOCK'S NEW MONTHLY MAGAZINE" has been received. It is a large, handsomely printed and beautifully illustrated magazine, devoted to Dramatic, Musical, and Art Notes. It should find a place in every family, as nothing tends more to refinement than a love for the Arts treated upon by this journal. Address: Benj. W. Hitchcock, No. 24 Beekman Street, N. Y., inclosing 25 cents for a number.

ZELL'S ENCYCLOPEDIA.—We notice this publication at every issue, because we like it. We advise every one to subscribe for it. It is a universal dictionary and one that is useful to all. T. Elwood Zell, publisher Phila, Pa.

PETERSON'S LADIES' MAGAZINE.—Ladies, take our advice, forward $2.00 to Charles J. Peterson, at 306 Chesnut Street, Phila., and receive the cheapest and best ladies' magazine in this country. Large inducements are offered to those getting up clubs for this journal. Send for a specimen number and learn what premiums are offered.

ADVENTURES IN THE WILDERNESS OR *Camp Life in the Adirondacks.*—This little volume is universally well spoken of. To one who has visited this wild country of the Adirondacks, it revives the incidents of his own experience. And even if one has not already seen the places or met with any of the scenes which Mr. Murray describes so graphically, the book cannot fail to excite an earnest desire to be a sharer in some of his exciting adventures. He leads the reader of his book on into a realizlng sense of the enjoyments of a sporting trip. We share with him the sportman's triumphs and the sportsmans toil, we see the mountains and streams, we enjoy the midnight bivouac after our adventurous tramp. If you cannot go yourself, to partake of the next best thing—get the book which tells you of it.

THE "RAGGED DICK STORIES."—A series of four volumes are most welcome books to young folks. In them is illustrated the life and experiences of the friendless and vagrant children who throng New York by thousands. The characters are taken from life and made to exhibit the trials and privations which these little folks are called upon to suffer. There are many unfortunate boys who go on to worthlessness because they have no way to get above the lot which has befallen them. Naturally quick and retentive of all which they lay hold of, their contact with the degrading, serves to draw them down instead of elevating them. Like many of the characters which Dickens has immortalized, these of Mr. Algers are true. They are not one whit less worthy of study. These books ought to be in the hands of all the boys of these wide awake times.

THE ILLUSTRATED LIBRARY OF WONDERS contains in a more than usually interesting style, a vast amount of useful information of many scientific wonders. Science is such a difficult thing to draw any interest from, that the help which these three little volumes bring is truly great. Beautiful illustrations explain to the eye, the facts which words may not have described. Already many scientific teachers, as well as those not deeply learned, are testifying to their worth. We have not seen a single person, as yet, who has looked at the beautiful pages of any of the volumes bearing respectively upon "*Thunder and Lightning*," "Optics," and "Wonders of Heat," who will not heartily endorse all we say of either. Old folks and young folks can find everything to please in their pages.

G. F. A.—*Latrobe, Pa.*—The work mentioned treats the subject scientifically. Is only for physicians use. Is not what you want.

H. J. O.—*Augusta, La.*—We can furnish back numbers of volume V, only. We have sent them you. We credit all subscriptions from the beginning of the volume.

DR. S.—*Lavonia, N. Y.*—You can procure the necessary instruments in New York or Phila.
A good amputating case will cost from $35 to $60.

L. A, M.—*Arcade, N. Y.*—It is the fault of a certain, secret, bad habit. Quit it or it will destroy your health and happiness.

DR. L.—*Phillipsburgh. Pa.*—The book can be had of Messrs Wood & Co., N. Y. Price $7.00.

MRS. A.—*Owego, N. Y.*—Babcock's uterine supporter is the best. See advertisement.

W. A. R.—*Brooklyn, N. Y.*—He's a "dead-beat." Was in the photograph business in this city and swindled everybody with whom he had dealings. Look out for him.

MRS. A.—*Port Jervis, N. Y.*—It is the fault of your frequent miscarriages. Our impression is that they are purposely produced. Quit it if you desire to be well. Inclose stamp when you wish answer by mail.

DR. L.—*Lock Haven, Pa.*—The man is not too old for the operation for cataract. We have frequently operated on persons between 80 and 90.

DR. J.—*Harrington's Corners, N. Y.*—The *Med. and Surg. Reporter*, Phila,, price $5. per year, or the *Record*, published by William Wood & Co., N. Y., price $4. per year.

J. D. B.—*Livingston, Texas.*—The symptoms you give indicate cataract. Medicine will do you no good. Apply to some competent Oculist for an operation.

MRS. W,—*Hornellsville, N. Y.*—Keep babies eyes perfectly free from matter by bathing them freely with warm water and castile soap. Procure one ounce of rose water, in which dissolve one grain of sulphate zinc. Apply this to its eyes by means of a camel's hair brush, morning and evening.

DR. R.—*Pittston, Pa.*—You will find your answer in Medical Items and News.

